# miR397/Laccase Gene Mediated Network Improves Tolerance to Fenoxaprop-*P*-ethyl in *Beckmannia syzigachne* and *Oryza sativa*

**DOI:** 10.3389/fpls.2017.00879

**Published:** 2017-05-23

**Authors:** Lang Pan, Hongwei Zhao, Qin Yu, Lianyang Bai, Liyao Dong

**Affiliations:** ^1^College of Plant Protection, Nanjing Agricultural UniversityNanjing, China; ^2^Key Laboratory of Integrated Management of Crop Diseases and Pests – Nanjing Agricultural University, Ministry of EducationNanjing, China; ^3^Australian Herbicide Resistance Initiative (AHRI), School of Agriculture & Environment, University of Western Australia, PerthWA, Australia; ^4^Biotechnology Research Center, Hunan Academy of Agricultural SciencesChangsha, China

**Keywords:** *Beckmannia syzigachne*, fenoxaprop-*P*-ethyl-resistance, non-target-site resistance, miR397, Laccase, metabolism

## Abstract

Herbicide resistance can be either target-site or non-target-site based. The molecular mechanisms underlying non-target-site resistance (NTSR) are poorly understood, especially at the level of gene expression regulation. MicroRNAs (miRNAs) represent key post-transcriptional regulators of eukaryotic gene expression and play important roles in stress responses. In this study, the *miR397* gene from *Beckmannia syzigachne* (referred to as *bsy-miR397*) was functionally characterized to determine its role in regulating fenoxaprop-*P*-ethyl resistance. We showed that (1) *bsy-miR397* transcript level is constitutively higher in resistant than in sensitive *B. syzigachne* plants, whereas *bsy-Laccase* expression and activity show the opposite trend, and (2) *bsy-miR397* suppresses the expression of *bsy-Laccase* in tobacco, indicating that it negatively regulates *bsy-Laccase* at the transcriptional level. We found evidences that miR397/laccase regulation might be involved in fenoxaprop-*P*-ethyl NTSR. First, the rice transgenic line overexpressing OXmiR397 showed improved fenoxaprop-*P*-ethyl tolerance. Second, following activation of *bsy-Laccase* gene expression by CuSO_4_ treatment, fenoxaprop resistance in *B. syzigachne* tended to decrease. Therefore, we suggest that *bsy-miR397* might play a role in fenoxaprop-*P*-ethyl NTSR in *B. syzigachne* by down-regulating laccase expression, potentially leading to the enhanced expression of three oxidases/peroxidases genes to introduce an active moiety into herbicide molecules in Phase-2 metabolism. *Bsy-miR397*, *bsy-Laccase*, and other regulatory components might form a regulatory network to detoxify fenoxaprop-*P*-ethyl in *B. syzigachne*, supported by the differential expression of transcription factors and oxidases/peroxidases in the rice transgenic line overexpressing OXmiR397. This implies how down-regulation of a gene (laccase) can enhance NTSR. Our findings shed light on the daunting task of understanding and managing complex NTSR in weedy plant species.

## Introduction

Herbicide resistance can be achieved by target-site and non-target-site based mechanisms (Powles and Yu, 2010). Target-site resistance (TSR) can be easily investigated by detecting mutation or expression of the target molecule. Non-target-site resistance (NTSR) refers to any mechanisms not belonging to TSR (Délye, 2013). NTSR is common among weed species and leads to the formation of unpredictable resistant phenotypes (Délye, 2013; Yu and Powles, 2014). NTSR to herbicides encompasses a range of diverse mechanisms, including reduced herbicide penetration and translocation, enhanced herbicide metabolism, and protection against the collateral damage of herbicide action, and regulatory pathways (Délye, 2013). NTSR can be mediated via several mechanisms (Délye, 2013; Yu and Powles, 2014), and often under polygenic control (Busi et al., 2013; Gaines et al., 2014; Duhoux et al., 2015; Gardin et al., 2015). Nevertheless, most recent studies have focused on metabolic resistance (Gaines et al., 2014; Duhoux et al., 2015; Gardin et al., 2015; Pan et al., 2016), and very few of them have investigated the molecular mechanisms underlying NTSR.

Plants are subjected to biotic and abiotic stresses in their environment and develop adaptation by evolving complex networks of stress detection and signaling and response pathways that trigger both general and specific responses along with adjustment of the response (Vaahtera and Brosché, 2011). Herbicides represent a powerful abiotic stress and are expected to trigger some of these pathways. Therefore, NTSR is assumed to be a part of weed stress response (Délye, 2013), and some of the herbicide-induced stress responses could be involved in NTSR. The regulation of plant response to stress involves multiple factors; NTSR is achieved by not only activating protection reactions but also via modifying regulators such as microRNAs (miRNAs) (Sunkar et al., 2012; Délye, 2013). As NTSR poses a high risk to crop production (Délye, 2013; Yu and Powles, 2014), determining the possible role of miRNAs in regulating herbicide response/resistance is necessary.

MicroRNAs are non-coding RNAs that are approximately 20–24 nucleotides long and are key posttranscriptional regulators of eukaryotic gene expression (Llave et al., 2002). They down-regulate the expression of target genes primarily by inhibiting or repressing their translation and are known to play important roles in adaptive responses to abiotic stresses (Khraiwesh et al., 2012). The first evidence of such roles for miRNA was found in *Arabidopsis thaliana* subjected to abiotic stress conditions, showing miRNAs different from those found in *A. thaliana* grown under normal conditions (Jones-Rhoades and Bartel, 2004; Sunkar and Zhu, 2004). To date, many miRNAs have been identified, and some of them have been experimentally confirmed to be related to various abiotic stress responses.

American sloughgrass (*Beckmannia syzigachne* Steud.) is an annual winter grass widely distributed in China. Since the 1990s, fenoxaprop-*P*-ethyl has been extensively and continuously utilized to control grass weeds, including *B. syzigachne*. Consequently, many *B. syzigachne* populations have evolved resistance to fenoxaprop-*P*-ethyl, thereby rendering them as hard-to-control weeds (Pan et al., 2015b). In our previous studies, three fenoxaprop-*P*-ethyl-resistant *B. syzigachne* populations involving both TSR and NTSR were identified (Pan et al., 2015b), and our RNA-seq analysis detected structural changes and alterations in the transcriptional regulation of several genes encoding metabolizing enzymes (Pan et al., 2016). The RNA-seq analysis also showed that laccase gene expression was down-regulated in the three fenoxaprop-*P*-ethyl-resistant *B. syzigachne* populations. However, we found no difference in laccase gene expression in several *B. syzigachne* populations exhibiting only TSR-based fenoxaprop-*P*-ethyl-resistance, compared to a susceptible population. All these results indicate that alterations in laccase gene transcriptional regulation might play a role in NTSR.

Since few studies have investigated laccase family members, and their potential biological roles have not been completely understood. In *A. thaliana*, laccases are known to play a role in lignin biosynthesis, and 17 laccase members that have various expression patterns and functions have been identified. Some of these laccase genes are associated with lignin biosynthesis (Lu et al., 2013); however, the roles of the other laccase genes remain unknown (Liang et al., 2006; Berthet et al., 2011). Furthermore, many laccase family members have been identified in rice, but their functions are unclear. Notably, studies have indicated that a laccase family member is a target of miR397 (Jones-Rhoades and Bartel, 2004). MicroR397 (miR397) is conserved across most dicotyledon and monocotyledon species (Jones-Rhoades and Bartel, 2004). At present, studies on miR397 have focused on its role in abiotic stress response. For example, a study found that miR397 expression was up-regulated in *A. thaliana* by a range of abiotic stresses such as cold, drought, and high salinity (Sunkar and Zhu, 2004). Similarly, the expression of miR397 was induced in rice in response to drought stress (Zhao et al., 2007). However, after persistent drought treatment, the expression of miR397 was down-regulated (Zhou et al., 2010). Furthermore, miR397 was also found to be up-regulated under oxidative stress caused by copper deficiency (Abdel-Ghany and Pilon, 2008; Li et al., 2011). As herbicides such as fenoxaprop-*P*-ethyl can cause oxidative stress to plants, and the question arises as whether some miRNAs might be involved in herbicide resistance and endow herbicide NTSR.

In this study, we characterized miR397 expression in a susceptible versus three resistant *B. syzigachne* populations (involving both TSR and NTSR). We also utilized transgenic tobacco and rice plants to further characterize the role of miR397 in regulating plant resistance and tolerance to fenoxaprop-*P*-ethyl. Our results suggest that *bsy-miR397* play a role in fenoxaprop-*P*-ethyl NTSR in *B. syzigachne* by down-regulating laccase expression, leading to changes in oxidase/peroxidase genes. To our knowledge, this is the first case of how down-regulation of a gene (laccase) can enhance NTSR. Our study also shows that down-regulation of laccase by miRNA has a causative role on certain oxidase/peroxidase genes.

## Materials and Methods

### Plant Material and Growth Conditions

Three fenoxaprop-*P*-ethyl-resistant *B. syzigachne* populations possessing both NTSR and TSR and one susceptible population (S) were used in this study (Pan et al., 2016). These populations were collected from the following locations: S from Feidong in Anhui Province, where fenoxaprop-*P*-ethyl was never applied; R1 from Xinlucun in Jiangsu Province with 10 years of fenoxaprop-*P*-ethyl use history; R2 from Lijiacun in Jiangsu Province, with 6 years of fenoxaprop-*P*-ethyl use; and R3 from Guocun in Jiangsu Province, with 5 years of fenoxaprop-*P*-ethyl use. These locations were more than 118 km apart from each other.

In our previous studies, we only compared the differences in gene expression between one resistant and one susceptible population, which might yield few “false positive” alleles that show differential expression between populations, but irrelevant to NTSR. Therefore, in the present study, we used three fenoxaprop-*P*-ethyl-resistant populations with different genetic backgrounds to minimize “false positives.”

Seeds were planted in pots filled with a 2:1 (wt/wt) mixture of sand and loam soil (pH 5.6; organic matter content 1.4%). All pots were placed in a greenhouse at 20°C/15°C day/night under a photoperiod of 12 h/12 h.

Tobacco (*Nicotiana benthamiana*) plants were grown in vermiculite containing Murashige and Skoog salt nutritional liquid in a growth chamber (light:dark period of 12:12 h at 25°C:18°C). Six-week-old tobacco plants were used for *Agrobacterium tumefaciens*-mediated transient expression as described (Chen et al., 2008).

The rice (*Oryza sativa* L.) wild type (WT) and miR397-overexpressing transgenic (OXmiR397) plants were kindly provided by Prof. Yueqin Chen from the Sun Yat-sen University (Zhang et al., 2013). The details about the rice plants were described (Zhang et al., 2013). Rice plants were grown in the culture solution (Zhang et al., 2013) supplemented with fenoxaprop-*P*-ethyl in a glasshouse with a day/night cycle of 12 h/12 h at 30°C/24°C.

### Quantitative Reverse-Transcriptase Polymerase Chain Reaction of *bsy-miR397* and *bsy-Laccase* in *B. syzigachne*

From the *B. syzigachne* mRNA sequence library (PRJNA290808) and miRNA sequence library (PRJNA290416) established in our laboratory, the *B. syzigachne* laccase gene (accession number: KP952245) and the *B. syzigachne* miR397 (sequence: 5′-ATTGAGTGCAGCGTTGATGAA-3′) were detected, and they were named as *bsy-Laccase* and *bsy-miR397*, respectively.

In total 300 plants from each of the three populations were cultivated to the tillering stage in 3-inch pots at day/night temperature of 20°C/15°C under a photoperiod of 12 h/12 h. Two tillers were collected from each plant: one for resistance/susceptibility analysis and the other for seed production. Resistance/susceptibility tested to fenoxaprop-*P*-ethyl was tested at the recommended field dose (62.0 g a.i.⋅ha^-1^), and survival assessed 3 weeks after treatment. The confirmed resistant tillers were placed in a pollen mask to produce progenies. In total, 15 susceptible and 285 resistant individuals were confirmed in R1 population; 15 susceptible and 285 resistant individuals in R2 population; and 16 susceptible and 284 resistant individuals in R3 population. The resistance tillers were self-pollinated in isolation to produce seeds of progeny populations. Thus, three resistance-enriched populations (R1, R2, and R3) were generated (Pan et al., 2016). With this method, three “purified” progeny R populations were obtained (thus minimizing genetic variation unrelated to fenoxaprop-*P*-ethyl resistance).

Plants from each population were cultivated up to the 3- to 4-leaf stage under the experimental conditions described above. Fenoxaprop-*P*-ethyl was applied at one-eighth of the recommended dose to allow survival of susceptible plants for gene expression analysis (Pan et al., 2016). The expression of candidate genes was measured in 30 untreated and 30 fenoxaprop-*P*-ethyl-treated plants for each fenoxaprop-*P*-ethyl dose at 24, 48, and 72 h. Total RNA was extracted using TRIzol (Invitrogen Inc., Carlsbad, CA, United States) following the manufacturer’s protocol. The quality and purity of RNA were assessed using the Nanodrop 2000 spectrophotometer (Thermo Scientific, Wilmington, DE, United States). Each RNA sample (10 μg) was subjected to reverse transcription using the Prime Script RT Reagent Kit (TaKaRa, Dalian, China) to produce cDNA. Small RNAs were reverse transcribed to cDNA using the Prime Script^TM^ miRNA qPCR Starter Kit (Version 2.0, TaKaRa, Dalian, China). The absence of gDNA contamination was confirmed by lack of amplification of the reference gene glyceraldehyde-3-phosphate dehydrogenase (GAPDH) using the primers containing an intron sequence.

Real-time PCR was performed using the ABI-7500 Fast Real-Time PCR System (ABI, United States) by using the SYBR^®^ *Premix Ex Taq*^TM^ kit (TaKaRa, Japan).

The relative expression of genes to that of the control was calculated using the 2^-ΔΔ*CT*^ method (Livak and Schmittgen, 2001). In each experiment, qPCR was conducted on each single plant (creating 30 data points) and repeated at least twice (two technical replicates). Significant differences in expression levels were analyzed using Welch’s *t*-test (Zheng et al., 2013). Two threshold values—a significant result in the *t*-test (*P <* 0.05) and a two-fold change were used to determine either up- or down-regulation. Capsine phosphatase, GAPDH, and ubiquitin were used as internal controls. The qPCR primers were designed based on the *bsy-Laccase* and *bsy-miR397* sequences. The primers used for qRT-PCR are listed in Supplementary Tables S1, S2. The reverse primer 5′-GTGCAGGGTCCGAGGT-3′ was used for *bsy-miR397* amplification.

We also included three populations (possessing TSR and in which metabolic resistance might not be involved) for comparison. These three populations were JYDX-R, SHQP-R, and JYSC-R. ACCase sequencing results showed that all plants in JYDX-R contained the Ile-1781-Leu mutation, those in SHQP-R contained the Ile-2041-Asn mutation, and those in JYSC-R contained the Gly-2096-Ala mutation. Next, 50 randomly selected plants from each of these populations were treated with fenoxaprop-*P*-ethyl + piperonyl butoxide (PBO, a known P450 inhibitor), and 20 randomly selected plants were treated with fenoxaprop-*P*-ethyl alone. No phenotypic differences were observed between plants subjected to fenoxaprop-*P*-ethyl alone and fenoxaprop-*P*-ethyl + PBO. When the three populations were compared with the S population, no difference was found in the structural and expressions of the 15 genes related to metabolic resistance. Our previous studies showed that PBO can partially reverse fenoxaprop resistance and that the 15 genes played a role in metabolic resistance in the three resistant populations R1, R2, and R3 (Pan et al., 2016); thus, populations JYDX-R, SHQP-R, and JYSC-R likely exhibited only TSR. The expression profiles of *bsy-miR397* and *bsy-Laccase* in JYDX-R, SHQP-R, and JYSC-R populations were analyzed as described above.

### Laccase Extraction and Assay

Laccase was isolated using the protocol of Wang et al. (2004) with slight modifications. Leaf samples of the S, R1, R2, R3 and JYDX-R, SHQP-R, JYSC-R plants were harvested at the 3- to 4-leaf stage. Crude laccase was extracted from the leaf material (200 mg fresh weight) in 1 mL buffer (25 mM MOPS, 200 mM CaCl_2_, pH 7.0), followed by centrifugation (16,000 × *g*, 10 min). Ten microliters of crude enzyme (about 6 μg protein) was added to a 0.5-mL mixture, and the reaction was conducted at 30°C for 30 min. Coomassie brilliant blue method was used to quantify the (total) protein using bovine serum albumin as the standard. Laccase activity prior to and after fenoxaprop treatment (24, 48, and 72 h) was determined colorimetrically by measuring the oxidation rate of 2 mg/mL 2,2-azobis (3-ethylbenzothiazoline-6-sulphonic acid) at 420 nm. The test was performed twice with three replicates per treatment.

### Co-expression of *bsy-miR397* and *bsy-Laccase* in Tobacco

The *bsy-miR397* and *bsy-Laccase* co-transformation in tobacco leaves was used to validate the interaction between *bsy-miR397* and *bsy-Laccase* genes. A full-length (1,740 bp) cDNA of *bsy-Laccase* was RT-PCR amplified from *B. syzigachne* by using the primers listed in Supplementary Table S3, and the sequence-confirmed PCR fragment was inserted into the pEarleyGate vector 202 under the control of a 35S promoter (Earley et al., 2006). The amiR-*bsy-miR397* was constructed using a previously described method (Warthmann et al., 2008). The primers for amiR-*bsy-miR397* amplification are listed in Supplementary Table S3. A 21-mer sequence was used to replace the endogenous miRNA and miRNA^∗^ in the plasmid pRS300 (Schwab et al., 2006) designed using the Web MicroRNA Designer platform. The resulting amiRNA was cloned into pEarleyGate vector 201 under the control of a 35S promoter. All constructs were sequenced to confirm the intended construction and designs. Descriptions of the amiRNAs, their target sequences, and genes are listed in Supplementary Table S4.

The *Agrobacterium* strain GV3101 was used to transfect the recombinant vectors into tobacco leaf cells. The strains containing different recombinant vectors (35S:*bsy-miR397*, 35S:*bsy-Laccase*, and 35S:*Os-miR164-control*) were cultured to OD_600_ = 0.8 at 28°C in a shaker at 220 rpm. Each cultured sample was collected and diluted to OD_600_ = 0.5 with MgCl_2_ and acetosyringone. The 35S:*bsy-laccase* and 35S:*bsy-miR397* samples were mixed in equal volumes, and the mixture was adjusted to OD_600_ = 1.0 and used to test the cleavage function of *bsy-miR397*. In the tobacco assay, laccase needs to be co-expressed with other non-effect miRNAs as control. As *Os-miR164* has no effect on laccase, we co-expressed *Os-miR164* and laccase to test our hypothesis. Subsequently, 100 μL of each treatment sample was used to infiltrate the tobacco leaves. The agrobacteria were infiltrated into the tobacco leaves, and treated leaves were harvested 2 days later. Total RNAs were extracted for qRT-PCR. Tobacco 18S rRNA expression was used as an internal standard for normalization. Primers used to detect the expression of *bsy-Laccase* and *bsy-miR397* in these plants are listed in Supplementary Tables S1, S2.

### Sensitivity to Fenoxaprop-*P*-ethyl and Other Herbicides in OXmiR397-Overexpressing Rice

Wild type and OXmiR397-overexpressing transgenic rice seeds were planted in pots containing commercial potting soil (Zhang et al., 2013) and maintained in a well-watered state in a greenhouse. When the seedlings reached the 3- to 4-leaf stage, fenoxaprop-*P*-ethyl was applied at the recommended field dose (62 g a.i.⋅ha^-1^) to the WT and OXmiR397 rice lines. Whether miR397/laccase is involved specifically in fenoxaprop-*P*-ethyl resistance was determined by including another ACCase-inhibiting herbicide haloxyfop-*R*-methyl (recommended field dose, 40.5 g a.i.⋅ha^-1^). Three weeks after treatment, the root length, plant height, and fresh weight were determined for each plant.

The target for OXmiR397 in rice is LOC_Os05g38420 (*OsLAC*) (Jeong et al., 2011; Zhang et al., 2013). The *OsLAC* expression and laccase activity were quantified in rice as described above. The primers were *OsLAC*-F (5′-GAGGAGGTGCCCATCATGTTC-3′) and *OsLAC*-R (5′-CCTTCAGCTTAAACGTGTCTTGG-3′) were used for quantification of *OsLAC* expression.

### *B. syzigachne* Sensitivity to Fenoxaprop-*P*-ethyl Following Treatment with 20 mM CuSO_4_

Laccase expression has been shown to be induced by 20 mM CuSO_4_ (Zhu and Ding, 2003; Jin et al., 2008). This approach was also used to determine the role of miR397/laccase in fenoxaprop-*P*-ethyl resistance in *B. syzigachne*. Induction of the *bsy-Laccase* expression in *B. syzigachne* by 20 mM CuSO_4_ was confirmed by comparing the expression level of *bsy-Laccase* with and without CuSO_4_ treatment.

Seeds from the S and three *B. syzigachne* populations possessing both TSR and NTSR were germinated using the method described previously (Pan et al., 2015b). The 3-week-old (at the 3- to 4-leaf stage) plants were treated with fenoxaprop-*P*-ethyl alone, and 20 mM CuSO_4_ plus fenoxaprop-*P*-ethyl using a laboratory sprayer as described previously (Pan et al., 2015b). The experiments were conducted twice in a completely randomized design with four replicates per treatment. Herbicide application rates were selected around the GR_50_ value of each population. Fenoxaprop-*P*-ethyl was applied at 0, 5, 10, 20, 40, 80, and 160 g a.i. ha^-1^ to the susceptible population (S) and at 0, 80, 160, 320, 640, 1280, and 2560 g a.i. ha^-1^ to the resistant populations (R1, R2, and R3). CuSO_4_ was applied 1 h before fenoxaprop-*P*-ethyl application. Three weeks after treatment, the above-ground material was harvested, and fresh weights were determined.

The experiments with fenoxaprop-*P*-ethyl and 20 mM CuSO_4_ plus fenoxaprop-*P*-ethyl treatments in JYDX-R, SHQP-R, and JYSC-R populations were conducted as described above.

### RNA-Seq Data Processing and Differentially Expressed Transcription Factor Analysis in *B. syzigachne*

MiR397 has been implicated in the network for lignin biosynthesis via putative interactions with several transcription factors (TFs) and laccases in *Populus trichocarpa* (Lu et al., 2013). The possible mechanism of miR397/laccase in regulating plant resistance to fenoxaprop-*P*-ethyl was assessed by constructing *B. syzigachne* RNA-seq libraries using RNA from the S population and three R populations (R1, R2, and R3) (Pan et al., 2016). The four libraries were assayed for quality and quantity and sequenced by Beijing BioMarker Technologies (Beijing, China). The mRNA-Seq libraries were constructed and sequence data were analyzed, assembled, and annotated as described by Pan et al. (2016). Gene names of specific TFs were assigned based on the best Basic Local Alignment Search Tool hit (highest score), excluding any uninformative descriptions.

The results of the comparison between S and R libraries suggested that RSEM could be used to quantify the reads identified as TFs (Li and Dewey, 2011). Transcript levels were quantified by counting reads per kilobase of exon model per million mapped reads (RPKM) (Mortazavi et al., 2008). The RPKM method of read density reflects the molar concentration of a transcript by normalizing for RNA length and the total number of reads. The EBSeq software was used to determine *P*-values for the samples with no biological replicates. EBSeq employs an empirical Bayes hierarchical model (Leng et al., 2013). A two-fold change, a *P*-value of <0.01, and a minimum RPKM of 2 in at least one of the treatments were used as the criteria to classify a response as up- or down-regulation. TFs were found to be consistently up- or down-regulated in R1, R2, and R3.

The downstream effect of laccase down-regulation on the up-regulated pathways that contribute to NTSR against fenoxaprop were further confirmed by conducting additional experiments that detected the expressions of these TFs and oxidase/peroxidase genes in WT and OXmiR397 rice. Primers used for detecting their expression are listed in Supplementary Table S5.

## Results

### Expression of *bsy-miR397* and *bsy-laccase* in *B. syzigachne*

**FIGURE 1:**
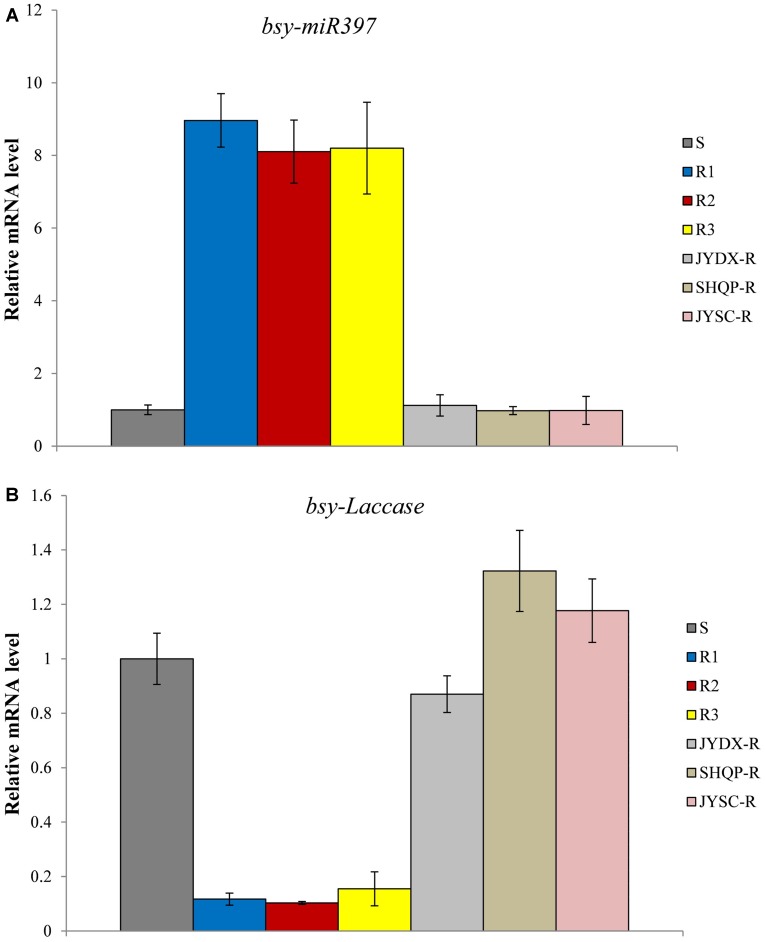
**The relative expression levels of *bsy-miR397* and *bsy-Laccase* in sensitive (S) and resistant (R) plants of *Beckmannia syzigachne*.** **(A)** *bsy-miR397*, **(B)** *bsy-Laccase*. qPCR was conducted on each single plant (creating 30 data points) and repeated at least twice (two technical replicates). Data are the mean values of these replicates. The standard errors of the means are described by vertical bars. S, collected from where fenoxaprop-*P*-ethyl was never applied; R1, R2, and R3, three fenoxaprop-*P*-ethyl-resistant populations possessing both NTSR and TSR; JYDX-R, SHQP-R, and JYSC-R, fenoxaprop-*P*-ethyl-resistant populations exhibited only TSR.

Quantitative PCR was used to analyze the expression of *bsy-miR397* and *bsy-Laccase* in 30 plants each of the S, R1, R2, R3, and JYDX-R, SHQP-R, JYSC-R population. Expression of *bsy-miR397* was significantly higher in the 30 plants each of the R1, R2, and R3 populations than in the S population, whereas the expression of *bsy-Laccase* was significantly higher in the S plants than in the R1, R2, and R3 plants (**Figure 1**). The expression of *bsy-miR397* was 8–9 times higher in plants of the R1, R2, and R3 populations than in the S population (**Figure 1**). In contrast, the expression of *bsy-Laccase* was 8–9-times lower in the plants of the R1, R2, and R3 populations than in the S population (**Figure 1**). Notably, we found that in JYDX-R, SHQP-R, and JYSC-R plants that likely exhibited only TSR, the expression patterns of *bsy-miR397* and *bsy-Laccase* were consistent with that in the S plants (**Figure 1**).

**FIGURE 2:**
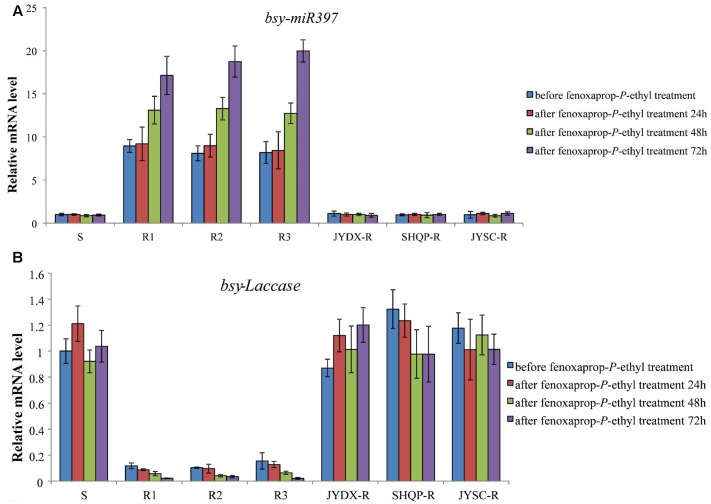
**The relative expression patterns of *bsy-miR397* and *bsy-Laccase* in sensitive (S) and resistant (R) plants of *Beckmannia syzigachne* before and after fenoxaprop treatment (24, 48, and 72 h).** **(A)** *bsy-miR397*, **(B)** *bsy-Laccase*. qPCR was conducted on each single plant (creating 30 data points) and repeated at least twice (two technical replicates). Data are the mean values of these replicates. The standard errors of the means are described by vertical bars.

The time-response expression profiles of *bsy-miR397* and *bsy-Laccase* were determined in 30 plants each of the S, R1, R2, R3 and JYDX-R, SHQP-R, JYSC-R populations at 24, 48, and 72 h after fenoxaprop-*P*-ethyl treatment. In the S and JYDX-R, SHQP-R, JYSC-R plants, the expression of *bsy-Laccase* and *bsy-miR397* remained unchanged (**Figure 2**). However, in the R1, R2, and R3 plants, after fenoxaprop-*P*-ethyl treatment, the expression of *bsy-miR397* was up-regulated, whereas *bsy-Laccase* down-regulated (**Figure 2**). This was consistent with the expression of these two genes in the untreated R1, R2, and R3 plants. These data indicate a negative correlation between *bsy-miR397* and *bsy-Laccase* expression both constitutively and in response to fenoxaprop-*P*-ethyl treatment in *B. syzigachne* R1, R2, and R3 populations.

### Laccase Activity in *B. syzigachne*

**FIGURE 3:**
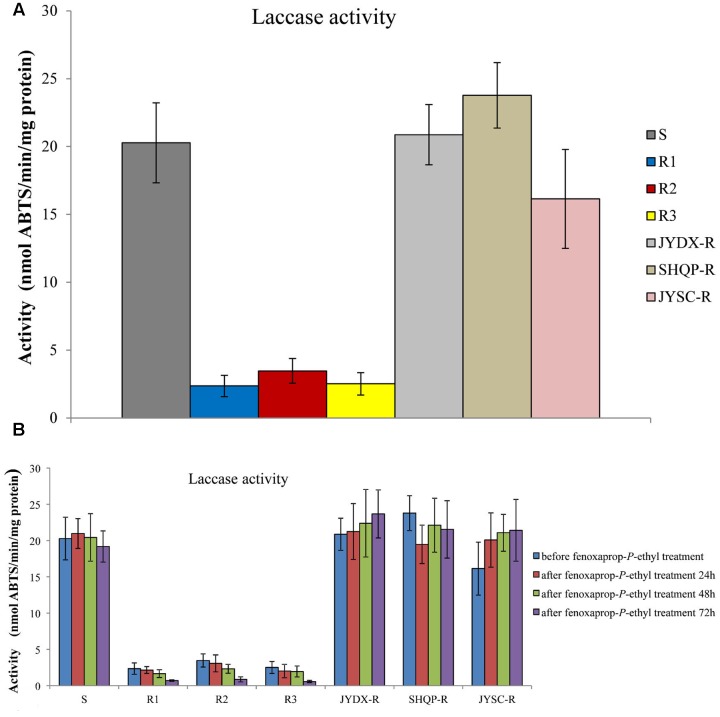
**Laccase activities in sensitive (S) and resistant (R) plants of *Beckmannia syzigachne* were determined by monitoring the oxidation (nmol 2,2-azobis [3-ethylbenzothiazoline-6-sulphonic acid]⋅min^-1^⋅mg^-1^ protein) of ABTS at 420 nm.** **(A)** Laccase activities before fenoxaprop treatment, **(B)** laccase activities before and after fenoxaprop treatment (24, 48, and 72 h). Data are the mean values of six biological replicates. The standard errors of the means are described by vertical bars.

Consistent with *bsy-Laccase* expression in *B. syzigachne*, a higher level (5.8- to 8.6-fold) of laccase activity was detected in the S than in R1, R2, and R3 plants (*P* < 0.05; **Figure 3A**). These results are consistent with those for *bsy-Laccase* gene expression, suggesting that laccase might play a role in fenoxaprop-*P*-ethyl resistance of *B. syzigachne*.

However, after fenoxaprop-*P*-ethyl treatment, laccase activity in R1, R2, and R3 plants decreased relative to that in the untreated R1, R2, and R3 plants (**Figure 3B**), whereas in the S plants laccase activity did not change considerably. We found that in JYDX-R, SHQP-R, and JYSC-R plants, laccase activity were consistent with that in the S plants either before or after fenoxaprop-*P*-ethyl treatment (**Figure 3**). These results are also consistent with the *bsy-Laccase* expression pattern in S and R1, R2, and R3 plants after fenoxaprop-*P*-ethyl treatment.

### *Bsy-Laccase* Expression Was Repressed by *bsy-miR397* in Tobacco

In rice, the expression of laccase genes is known to be regulated by miRNA397 (Zhang et al., 2013). The target gene of *bsy-miR397* is predicted to be *bsy-Laccase* from http://www.mirbase.org, and the 714- to 734-bp region on the *bsy-Laccase* gene might be a target site for *bsy-miR397*. Hence, we hypothesized that *bsy-Laccase* gene could be cleaved by *bsy-miR397*. Since co-expression in tobacco is one of the best ways to validate the interaction between an miRNA and its predicted target (Qi et al., 2012), we co-expressed the *bsy-miR397* and *bsy-Laccase* genes in tobacco.

Quantitative RT-PCR analyses revealed that the *bsy-Laccase* expression in tobacco carrying 35S:*bsy-Laccase*/35S:*bsy-miR397* was 3.6-fold lower than that in tobacco plants carrying only 35S:*bsy-Laccase* (Supplementary Figure S1). These results indicate that *bsy-Laccase* expression was repressed by *bsy-miR397* in transformed tobacco carrying 35S:*bsy-Laccase*/35S:*bsy-miR397*, and *bsy-Laccase* might be the target gene of *bsy-miR397*.

### Improved Tolerance to Fenoxaprop-*P*-ethyl in OXmiR397-Overexpressing Rice

As miR397 is conserved across most dicotyledon and monocotyledon species, and fenoxaprop-*P*-ethyl has no activity in broadleaf tobacco, we attempted to further investigate the role of miR397/laccase in fenoxaprop-*P*-ethyl resistance by using OXmiR397-overexpressing transgenic rice and the corresponding WT lines.

**FIGURE 4:**
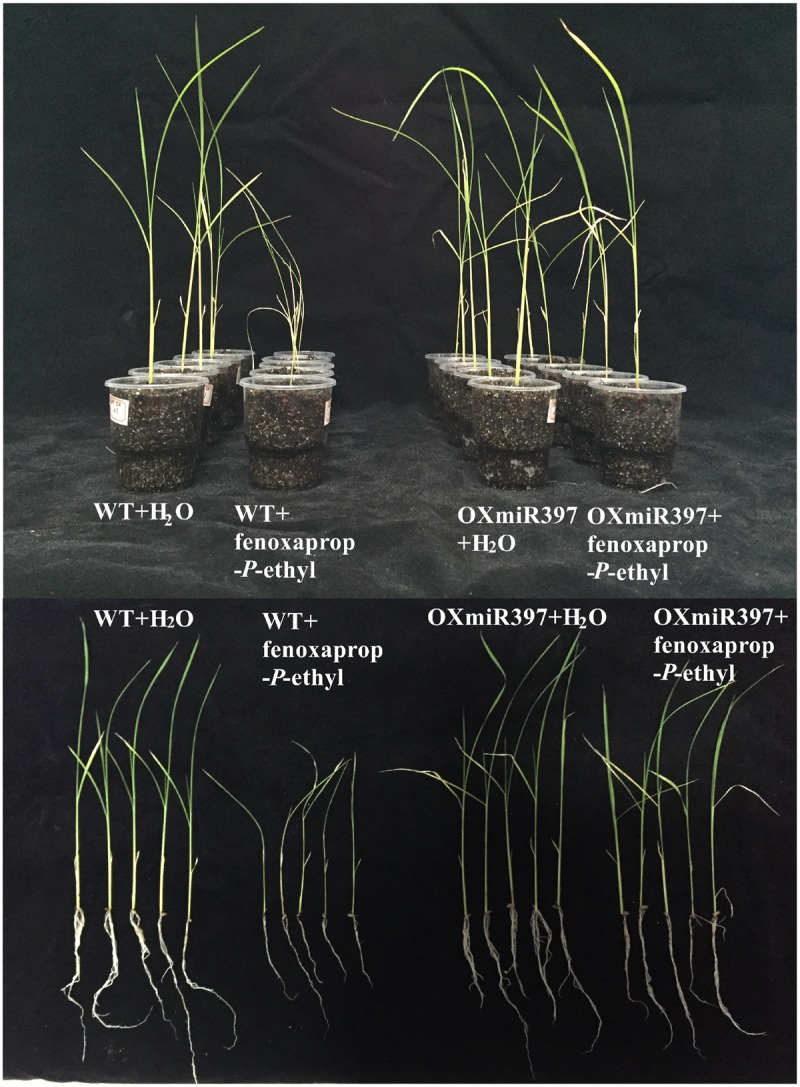
**Overexpression of rice miR397 gene improves fenoxaprop-*P*-ethyl tolerance in rice.** WT, wild type.

**FIGURE 5:**
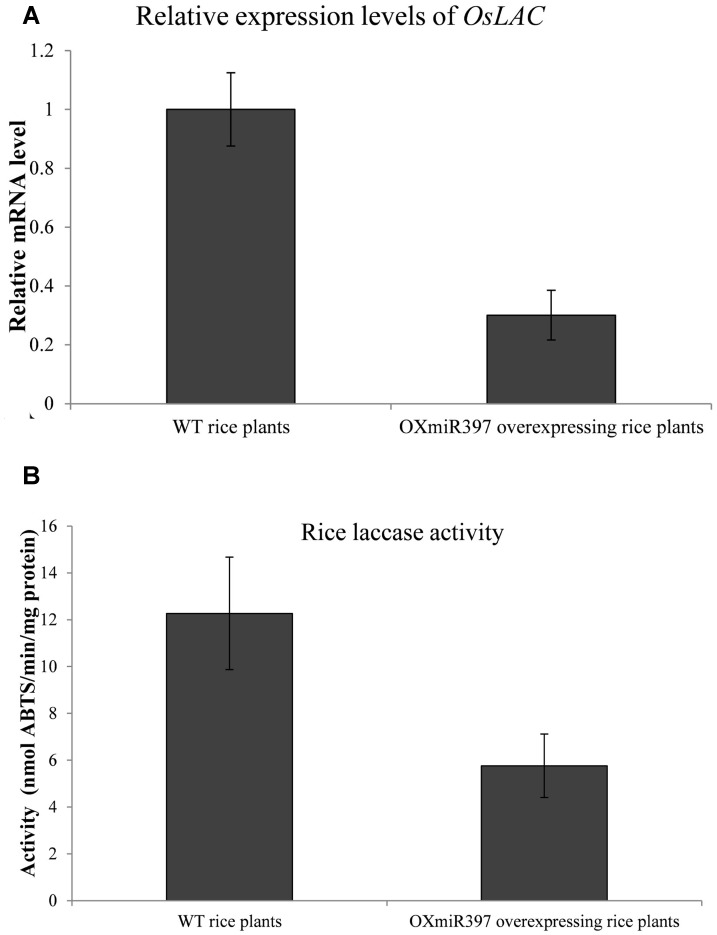
**Relative expression levels of *OsLAC* mRNA** **(A)** and laccase acitivity **(B)** in wild type (WT) and OXmiR397-overexpressing rice plants. qRT-PCR used three independent biological replicates and repeated at least twice (two technical replicates). Data are the mean values of six biological replicates. The standard errors of the means are described by vertical bars.

Rice OXmiR397 is known to regulate laccase. To determine if this regulation has an impact on herbicide tolerance, we treated WT and OXmiR397-overexpressing transgenic rice with the field rate of fenoxaprop-*P*-ethyl (62 g a.i. ha^-1^), with untreated rice plants as a control. Seven days after fenoxaprop-*P*-ethyl treatment, WT rice plants started to show phytotoxicity symptoms such as leaf withering and reduced plant height, whereas OXmiR397-overexpressing plants showed no visual damage. Three weeks after treatment, WT rice plants showed severe tissue damage, whereas OXmiR397-overexpressing plants remained largely healthy and green (**Figure 4**). In addition, OXmiR397-overexpressing plants exhibited significantly greater root length and plant height (**Figure 4** and **Table 1**) as well as overall fresh weight than the WT rice (**Figure 4** and **Table 1**). Six weeks after fenoxaprop-*P*-ethyl treatment, WT plants died, whereas OXmiR397-overexpressing plants only showed slight tissue damage (which occurred about a week later than the WT plants and never recovered). As expected, OXmiR397-overexpressing plants had lower levels of *OsLAC* expression and laccase activity than the WT plants (**Figure 5**). These results indicated that miR397/laccase regulation can also provide protection from fenoxaprop-*P*-ethyl damage in OXmiR397-overexpressing transgenic rice, suggesting that miR397/laccase might be involved in fenoxaprop-*P*-ethyl NTSR.

**Table 1:** Root length, plant height, and fresh weight in OXmiR397 overexpressing and WT rice plants treated with fenoxaprop (at the recommended field dose).

	Root length^∗^ (cm) (SE)	Plant height^∗^ (cm) (SE)	Fresh weight^∗^ (g) (SE)
WT + H_2_O	16.2 (1.30)	30.9 (1.88)	0.36 (0.053)
WT + fenoxaprop	9.7* (2.77)	19.0* (1.37)	0.11* (0.016)
OXmiR397 + H_2_O	15.1 (2.66)	32.4 (1.50)	0.38 (0.049)
OXmiR397 + fenoxaprop	14.2 (2.05)	29.5 (2.35)	0.35 (0.052)


*^∗^Significant difference (*P* < 0.05) from GenePattern analysis. SE, standard error.*

In contrast, 3 weeks after treatment with another ACCase herbicide haloxyfop-*R*-methyl at the recommended field dose, OXmiR397-overexpressing rice and WT plants all exhibited significantly smaller root length and plant height than that without haloxyfop-*R*-methyl treatment (**Table 2**). Six weeks after treatment, they all died. This result suggested that the miR397/laccase pathway might not be a general defense mechanism against all herbicides, but likely specific to fenoxaprop-*P*-ethyl.

**Table 2:** Root length, plant height, and fresh weight in OXmiR397 overexpressing and WT rice plants treated with haloxyfop (at the recommended field dose).

	Root length^∗^ (cm) (SE)	Plant height^∗^ (cm) (SE)	Fresh weight^∗^ (g) (SE)
WT + H_2_O	16.4 (0.82)	31.3 (1.52)	0.37 (0.027)
WT + haloxyfop	8.3* (1.57)	18.7* (1.01)	0.10* (0.015)
OXmiR397 + H_2_O	15.3 (0.91)	31.8 (1.35)	0.36 (0.044)
OXmiR397 + haloxyfop	7.6* (0.89)	18.1* (1.52)	0.11* (0.020)


*^∗^Significant difference (*P* < 0.05) from GenePattern analysis. SE, standard error.*

### Impact of 20 mM CuSO_4_ on Fenoxaprop-*P*-ethyl Resistance in *B. syzigachne*

**FIGURE 6:**
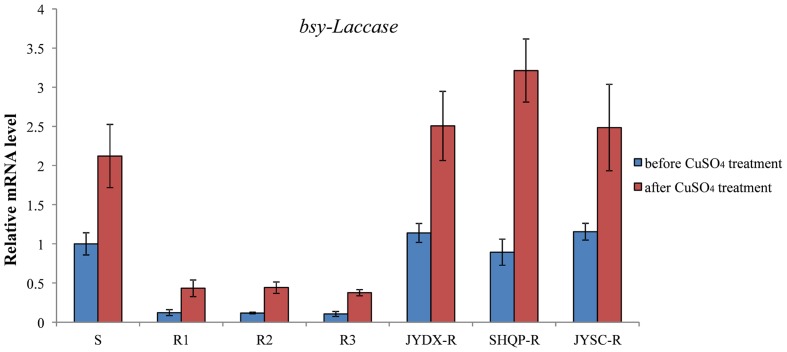
**The expression level of *bsy-Laccase* before and after CuSO_4_ treatment in *B. syzigachne*.** Data are the mean values of replicates. The standard errors of the means are described by vertical bars.

In this experiment, 3-week-old (at the 3- to 4-leaf stage) *B. syzigachne* plants were used. Treatment with 20 mM CuSO_4_ alone did not have a visual effect on *B. syzigachne* plants (**Table 3**). However, *bsy-Laccase* expression was significantly induced 1 h after CuSO_4_ treatment in all populations (**Figure 6**). Fenoxaprop-*P*-ethyl treatment in combination with 20 mM CuSO_4_ increased herbicide toxicity in the three *B. syzigachne* R populations, with a slight (but significant) reduction of the GR_50_ (the effective rate of herbicide causing 50% inhibition in plant fresh weight) value (**Table 3**), whereas the GR_50_ value of the S population did not change significantly. These results indicate that the activation of *bsy-Laccase* by CuSO_4_ in fenoxaprop-*P*-ethyl-resistant *B. syzigachne* can have an impact on resistance.

**Table 3:** Effects of fenoxaprop-*P*-ethyl and 20 mmol L^-1^ CuSO_4_ on growth of resistant R1, R2, and R3 *Beckmannia syzigachne* plants.

Treatments	% Inhibition of fresh weight (SE)				GR_50_ values (g a.i. ha^-1^) (SE)^b^			
		
	S	R1	R2	R3	S	R1	R2	R3
20 mmol/L CuSO_4_	1.32 (0.17)	0.21 (0.07)	1.11 (0.14)	0.32 (0.04)	–	–	–	–
Fenoxaprop-*P*-ethyl	–	–	–	–	44.8 (3.27)	2412 (35.9)	2029 (47.9)	1217 (37.3)
Fenoxaprop-*P*-ethyl + 20 mmol/L CuSO_4_	–	–	–	–	43.8 (8.31)	1876^∗^ (30.4)	1539^∗^ (38.4)	920^∗^ (49.9)


*^b,∗^ Difference between fenoxaprop-*P*-ethyl treatment and fenoxaprop-*P*-ethyl + 20 mmol/L CuSO_4_ treatment was tested for each population with an independent sample *t*-test. SE, standard error.*

However, CuSO_4_ treatment did not increase fenoxaprop-*P*-ethyl toxicity in JYDX-R, SHQP-R, and JYSC-R populations which mainly exhibited TSR (**Table 4**), indicating that laccase might play a role in *B. syzigachne* R1, R2, and R3 populations.

**Table 4:** Effects of fenoxaprop-*P*-ethyl and 20 mmol L^-1^ CuSO_4_ on growth of JYDX-R, SHQP-R, JYSC-R *Beckmannia syzigachne* plants.

Treatments	GR_50_ values (g a.i. ha^-1^) (SE)			
	
	S	JYDX-R	SHQP-R	JYSC-R
Fenoxaprop-*P*-ethyl	44.8 (3.27)	1251 (79.5)	1387 (29.8)	1118 (71.4)
Fenoxaprop-*P*-ethyl + 20 mmol/L CuSO_4_	43.8 (8.31)	1218 (53.4)	1427 (50.2)	1208 (39.8)


*SE, Standard error.*

### Differentially Expressed TF Analysis in *B. syzigachne*

From the RNA-seq libraries established in our previous study (Pan et al., 2016), we found 587 genes that were annotated as TFs. Of these, four TFs were consistently down-regulated in R1, R2, and R3, whereas the rest of them did not exhibit consistent changes in expression. The expression of two myeloblastosis (MYB) family genes (*bsy-MYB39* and *bsy-MYB2*) and two auxin response factor (ARF) genes (*bsy-ARF5* and *bsy-ARF8*) remained significantly lower in the R1, R2, and R3 plants than in the S plants (**Table 5**). In particular, the expression of *bsy-MYB39, bsy-MYB2*, *bsy-ARF5*, and *bsy-ARF8* was about 3–6 times lower in the R than in the S plants (**Table 5**).

**Table 5:** Identification of TF (transcription factors) genes down-regulated in resistant (R1, R2, and R3) and susceptible (S) *Beckmannia syzigachne* plants using the RPKM method for fold-change.

Gene	Contig	Fold-change (R1 value/S value)^∗,a^	Fold-change (R2 value/S value)^∗,a^	Fold-change (R3 value/S value)^∗,a^
*bsy-MYB39*	comp112703	-5.0^∗^ (4.6/23.1)	-3.9^∗^ (5.9/23.1)	-3.3^∗^ (7.0/23.1)
*bsy-MYB2*	comp12870	-4.4^∗^ (6.5/28.7)	-4.8^∗^ (6.0/28.7)	-6.4^∗^ (4.9/28.7)
*bsy-ARF5*	comp25306	-3.2^∗^ (17.8/57.6)	-3.8^∗^ (15.0/57.6)	-5.8^∗^ (10.0/57.6)
*bsy-ARF8*	comp10726	-3.9^∗^ (3.9/15.3)	-4.4^∗^ (3.5/15.3)	-4.5^∗^ (3.4/15.3)


*^∗^Significant difference (*P* < 0.05) from GenePattern analysis.*

*^a^ “-” Indicates that genes in the R lines were down-regulated compared with genes in the S lines. RPKM, reads per kb of exon model per million mapped reads.*

**FIGURE 7:**
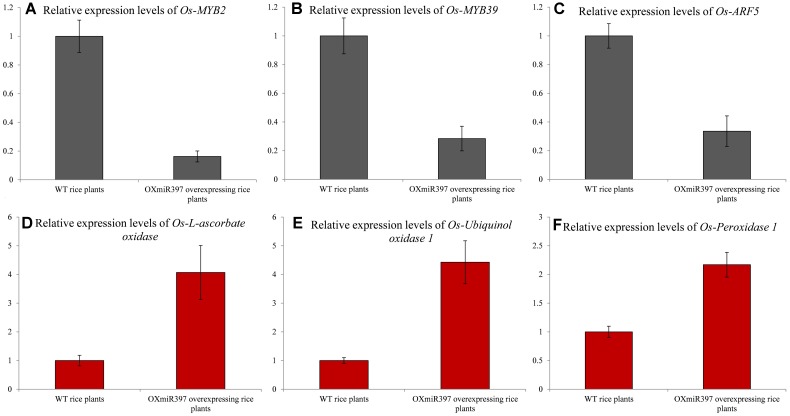
**The relative expression levels of the three transcription factors and three oxidase/peroxidase genes consistent with *B. syzigachne* in wild type (WT) and OxmiR397 rice.** **(A)** *Os-MYB2*, **(B)** *Os-MYB39*, **(C)** *Os-ARF5*, **(D)** *Os-l-ascorbate oxidase*, **(E)** *Os-Ubiquinol oxidase 1*, **(F)** *Os-Peroxidase*. qRT-PCR used three independent biological replicates and repeated at least twice (two technical replicates). Data are the mean values of six biological replicates. The standard errors of the means are described by vertical bars.

To further confirm the downstream effect of laccase down-regulation on the up-regulated pathways that contributes to NTSR against fenoxaprop, we analyzed the expression of the four TFs and four oxidase/peroxidase genes in WT and OXmiR397 rice. The results showed that the expression of *Os-peroxidase 66* and *Os-ARF8* remained unchanged between WT and OXmiR397 rice. However, the expression levels of *Os-ARF5*, *Os-MYB2*, and *Os-MYB39* were significantly lower in the OXmiR397 rice, whereas those of *Os-l-ascorbate oxidase*, *Os-ubiquinol oxidase 1*, and *Os-peroxidase 1* were significantly higher (**Figure 7**). In particular, the expression of *Os-MYB2* (**Figure 7A**), *Os-MYB39* (**Figure 7B**), and *Os-ARF5* (**Figure 7C**) was, respectively, 6.2, 3.5, and 3.0 times lower in OXmiR397 rice than in WT rice. In contrast, the expression of *Os-l-ascorbate oxidase* (**Figure 7D**), *Os-ubiquinol oxidase 1* (**Figure 7E**), and *Os-peroxidase* (**Figure 7F**) was, respectively, 4.1-, 4.4-, and 2.2-fold higher in OXmiR397 rice than in WT rice.

The relative expression levels of these three TFs and three oxidase/peroxidase genes are consistent with those in *B. syzigachne*, indicating that ARF5, MYB2, MYB39, l-ascorbate oxidase, ubiquinol oxidase 1, and peroxidase 1 might be involved in the regulatory network with *bsy-miR397*/*bsy-Laccase*.

## Discussion

Several studies have revealed that miR397 has pivotal roles in plant responses to diverse abiotic stresses including H_2_O_2_, cold, dehydration, NaCl, and abscisic acid (Sunkar and Zhu, 2004; Luo et al., 2006). However, no previous studies have investigated the contribution or underlying molecular mechanisms of miR397 involvement in herbicide resistance. In this study, we experimentally confirmed that miR397 is likely involved in fenoxaprop resistance in *B. syzigachne* and in the improvement of fenoxaprop tolerance in rice via the negative regulation of laccases.

Laccases are known to act as targets of miR397 (Jones-Rhoades and Bartel, 2004). *Bsy-Laccase* was predicted to be a target gene of *bsy-miR397*, and our experiments performed using transient co-expression of *bsy-Laccase* and *bsy-miR397* in tobacco confirmed this possibility (Supplementary Figure S1). It is worth to note that *bsy-miR397* mediated suppression of *bsy-Laccase* gene in tobacco was only 3.6-fold. To increase the level of suppression to get increased NTSR, in the future we can try an inducible Gateway-compatible expression vector that allows tighter control of gene expression than previously designed inducible systems (Earley et al., 2006). This ‘double-lock’ inducible system remains sequestered in the cytoplasm until dexamethasone treatment, which allows the protein to move into the nucleus, catalyze the removal of the sequence blocking transcription by the 35S promoter, and thereby allow expression of the target gene (Joubès et al., 2004).

Laccases are four copper atom-containing glycoproteins that catalyze the oxidation of a suitable substrate molecule leading to the production of water and oligomers, including diphenol, dinaphthol, phenylenediamine, and anthranilate-derived metabolites (LaFayette et al., 1999). Cupric ions are present in the active sites of laccases. Cupric ions induce laccase activity by combining the free ω-carboxylic group anions of acidic amino acid residues around the active sites of laccases and affect the charge distribution during electron transfer (Zhu and Ding, 2003; Jin et al., 2008). Unlike for fungal laccases (Mayer and Staples, 2002), the roles of plant laccases are not yet completely understood, although they might be related to lignin biosynthesis (Ranocha et al., 2002) and copper homeostasis (Claus, 2004). They are required for the maintenance of copper homeostasis in many processes such as energy transduction and oxidative stress responses (Arredondo and Nunez, 2005). In turn, the down-regulation of laccases might limit some non-essential biological processes to allow the conservation of energy for defense purposes and to reserve required elements for the most essential functions under adverse stress conditions (Abdel-Ghany and Pilon, 2008). The activation of miR397 expression down-regulates laccase gene expression, suggesting that copper-responsive miRNAs reserve copper for more essential proteins such as cytochrome c oxidase (belonging to oxidases). Oxidases have been indicated to play an important role in NTSR for metabolizing herbicides (Délye, 2013).

The results of this study showed that the level of miR397 was higher in the resistant than in the susceptible *B. syzigachne* plants (**Figures 1**, **2**). Furthermore, transgenic rice plants overexpressing miR397 showed normal growth, whereas the untransformed WT plants showed severe symptoms (e.g., dwarfism and etiolation; **Figure 4**), following fenoxaprop-*P*-ethyl treatment. In addition, 20 mM CuSO_4_ was used to activate *bsy-Laccases* in *B. syzigachne* (**Figure 6**); consequently, the fenoxaprop resistance level decreased significantly (**Table 3**) owing to the up-regulated expression of *bsy-Laccase* (**Figure 6**). These results are in accord with the hypothesis proposed above that laccase down-regulation improves fenoxaprop tolerance via copper homeostasis/energy conservation. The application of CuSO_4_ induces the expression of laccase via this mechanism, thereby reducing essential oxidase proteins and down-regulating their expression, and finally reducing tolerance. These findings suggest that *bsy-miR397* and *bsy-Laccase* genes might play a role in fenoxaprop-*P*-ethyl resistance in *B. syzigachne* plants, and that increased levels of miR397 might be involved in the improvement of fenoxaprop-*P*-ethyl tolerance in rice.

Oxidases and peroxidases, such as those identified to be up-regulated in our previous RNA-seq analysis (Pan et al., 2016), might be involved in converting herbicide molecules into more hydrophilic metabolites, contributing to herbicide resistance (Délye, 2013). The relative abundance of laccases and peroxidases has been reported to be reciprocally regulated to mediate lignin polymerization in the *Ptr-MIR397a* regulatory networks, further contributing to the functional redundancy of enzymes for oxidative polymerization (Lu et al., 2013). Given that oxidases are important in NTSR, laccase and oxidases/peroxidases might also be reciprocally regulated to finally increase herbicide tolerance via the miR397 regulatory networks (Lu et al., 2013). In a previous RNA-seq analysis in the fenoxaprop-*P*-ethyl-resistant *B. syzigachne* populations used in the present study, we also found that two genes each in the oxidase gene family (*ubiquinol oxidase 1* and *l-ascorbate oxidase*) and peroxidase family (*peroxidase 1* and *peroxidase 66*) were constitutively up-regulated (Pan et al., 2016). In addition, four TFs (*bsy-MYB39*, *bsy-MYB2*, *bsy-ARF5*, and *bsy-ARF8*) were found to be consistently down-regulated in the resistant plants tested (R1, R2, and R3; **Table 5**). Previously, laccase has been implicated in the network for lignin biosynthesis by putative interactions with several TFs and peroxidases (Lu et al., 2013). In the present study, we also found that these TFs and oxidase/peroxidase genes were expressed in WT and OXmiR397-expressing rice. Their expression patterns are consistent with those in *B. syzigachne*, including ARF5, MYB2, MYB39, l-ascorbate oxidase, ubiquinol oxidase 1, and peroxidase 1 (**Figure 7**). As only miR397 was over-expressed in OXmiR397 rice, whereas all the other miRNAs showed the same expression as that in WT, these TFs and oxidase/peroxidase genes might participate in the miR397/laccase mechanism. Our findings indicated that the identified genes, including *bsy-miR397*, *bsy-Laccase*, and associated TFs and oxidases/peroxidases, might also form a regulatory network to detoxify fenoxaprop-*P*-ethyl in *B. syzigachne*.

Therefore, we hypothesized that in resistant *B. syzigachne* plants, *bsy-miR397* might be part of a regulatory network mediating fenoxaprop-*P*-ethyl resistance. In resistant *B. syzigachne*, *bsy-miR397* is expressed at a high level and thus down-regulates the abundance of laccases. This down-regulation of *bsy-Laccase* might regulate the downstream components of a network. *Bsy-Laccase* is implicated in such a network via the putative interactions with three TFs (*bsy-MYB39*, *bsy-MYB2*, and *bsy-ARF5*). As mentioned above, laccase down-regulation reserve energy for more essential oxidases proteins via copper homeostasis/energy conservation. These three TFs (*bsy-MYB39*, *bsy-MYB2*, and *bsy-ARF5*) might also control the up-regulation of three downstream pathway genes identified previously (*ubiquinol oxidase 1*, *l-ascorbate oxidase*, and *peroxidase 1*) (Pan et al., 2016). NTSR is largely conferred by an increase in the expression of oxidases/peroxidases to introduce an active moiety into herbicide molecules in Phase-2 metabolism (Délye, 2013). Therefore, the coordinated up-regulation of these three oxidase/peroxidase genes are likely involved in fenoxaprop-*P*-ethyl detoxification. The detected expression changes indicate the involvement of *bsy-miR397*/laccases and suggest *bsy-miR397* as a regulator in fenoxaprop-*P*-ethyl-resistant *B. syzigachne*. However, all of the proposed interactions require further experimental validation. Notably, the effect is evident only in response to fenoxaprop-*P*-ethyl and not to haloxyfop-*R*-methyl, suggesting that the *bsy-miR397*/laccases regulation mechanism might not be a general stress response. This need further investigation in the future.

In China, fenoxaprop-*P*-ethyl is the most widely used and highly efficient herbicide for controlling grass weeds in wheat fields. Its extensive and continuous use, often as the sole method of weed control, has resulted in the evolution of fenoxaprop-*P*-ethyl resistance in weeds. Accordingly, fenoxaprop-*P*-ethyl-resistant *B. syzigachne* has become one of the most predominant and troublesome weeds in wheat fields in China and has been reported in several regions of the country (Li et al., 2013, 2014; Pan et al., 2015a,b,c; Tang et al., 2015). However, although two classes of *GST* genes have been reported to be involved in fenoxaprop-*P*-ethyl resistance in black-grass (*A. myosuroides*) and ryegrass (*Lolium rigidum*) populations (Cummins et al., 1999, 2013), our understanding of NTSR to fenoxaprop-*P*-ethyl is limited. Elevated levels of P450 and GST enzymes have been reported in several fenoxaprop-*P*-ethyl-resistant black-grass populations (Reade and Cobb, 2002; Letouze and Gasquez, 2003). In addition, fenoxaprop resistance in black grass has recently been suggested to be caused by the scavenging peroxidase activities of specific GST enzymes as well as by the concomitant production of protective flavonoids to counteract the free noxious radicals generated by the herbicide action (Cummins et al., 2013).

In the three fenoxaprop-*P*-ethyl-resistant *B. syzigachne* populations (R1, R2, and R3) identified in this study, the resistance mechanisms to fenoxaprop-*P*-ethyl are diverse involving both TSR and multiple NTSR mechanisms. TSR to fenoxaprop-*P*-ethyl in this species is due to point mutations in the ACCase CT domain (Pan et al., 2015a), as with the other three populations JYDX-R, SHQP-R, and JYSC-R used in the present study. However, NTSR also exists in populations R1, R2, and R3 and is more complex, as we have detected structural changes and altered expression levels of 15 genes encoding metabolizing enzymes (Pan et al., 2016). In addition, *bsy-miR397* down-regulating laccase expression, as described in the present study, represents the first example of how down-regulation of a gene (laccase) can enhance NTSR in *B. syzigachne*. Knowledge of these resistance-regulating mechanisms is important for designing effective weed control strategies to manage and delay the onset of resistance to fenoxaprop-*P*-ethyl.

In summary, to our knowledge, this is the first report investigating how down-regulation of a gene (laccase) can enhance NTSR. We found that *bsy-Laccase* was inhibited by *bsy-miR397* via transient expression in tobacco. We also found that the overexpression of miR397 correlated with fenoxaprop-*P*-ethyl resistance in *B. syzigachne* and improved fenoxaprop-*P*-ethyl tolerance in rice. Therefore, we propose that *bsy-miR397* might play a role in NTSR to fenoxaprop-*P*-ethyl in *B. syzigachne* by down-regulating laccase expression, leading to associated changes in oxidase/peroxidase genes. The findings of this study thus indicated that NTSR might be driven by the manipulation of miR397/Laccase via the modulation of NTSR might be regulated by miR397/Laccase, and this may open a new avenue for understanding and managing complex NTSR in weedy plant species.

## Author Contributions

LP, QY, and LD designed the experiments. LP and HZ performed the experimental work. LP, HZ, LB and LD performed the data analysis. LD supervised the research and manuscript preparation. All authors wrote and edited the manuscript.

## Conflict of Interest Statement

The authors declare that the research was conducted in the absence of any commercial or financial relationships that could be construed as a potential conflict of interest.
