# Supplementary material for: miR397/Laccase Gene Mediated Network Improves Tolerance to Fenoxaprop-P-ethyl in Beckmannia syzigachne and Oryza sativa
Source: Front Plant Sci. 2017 May 23;8:879. doi: 10.3389/fpls.2017.00879 (PMC5440801; doi:10.3389/fpls.2017.00879)
Supplement: Supplementary file 1 [file Table_1.DOC]

*Supplementary Material*

**miR397/Laccase gene mediated network improves tolerance to fenoxaprop-*P*-ethyl in *Beckmannia syzigachne* and *Oryza sativa***

Lang Pan^1,2^, Hongwei Zhao^1,2^, Qin Yu^3^, Lianyang Bai^4^ & Liyao Dong^1,2^,*

^1^ *College of Plant Protection, Nanjing Agricultural University, Nanjing, China,*

^2^ *Key Laboratory of Integrated Management of Crop Diseases and Pests (Nanjing Agricultural University), Ministry of Education,* *Nanjing 210095, China,*

^3^ *Australian Herbicide Resistance Initiative (AHRI), School of Plant Biology, University of Western Australia, Crawley, 6009, Western Australia, Australia,*

^4^ *Biotechnology Research Center, Hunan Academy of Agricultural Sciences, Changsha 410125, China,*

*Correspondence*: Li-Yao Dong, College of Plant Protection, Nanjing Agricultural University, Nanjing 210095, Jiangsu, China. Tel: (+86) 025-84395672; Fax: (+86) 025-84395672; E- mail:* [*dly@njau.edu.cn*](mailto:dly@njau.edu.cn)

# Supplementary Figures and Tables

**Supporting Figure Legends**

**Figure S1. The relative expression level of *bsy-Laccase* in tobacco leaves measured using quantitative reverse-transcription polymerase chain reaction**. Coexpression of *bsy-miR397* and *bsy-Laccase* in tobacco leaves.


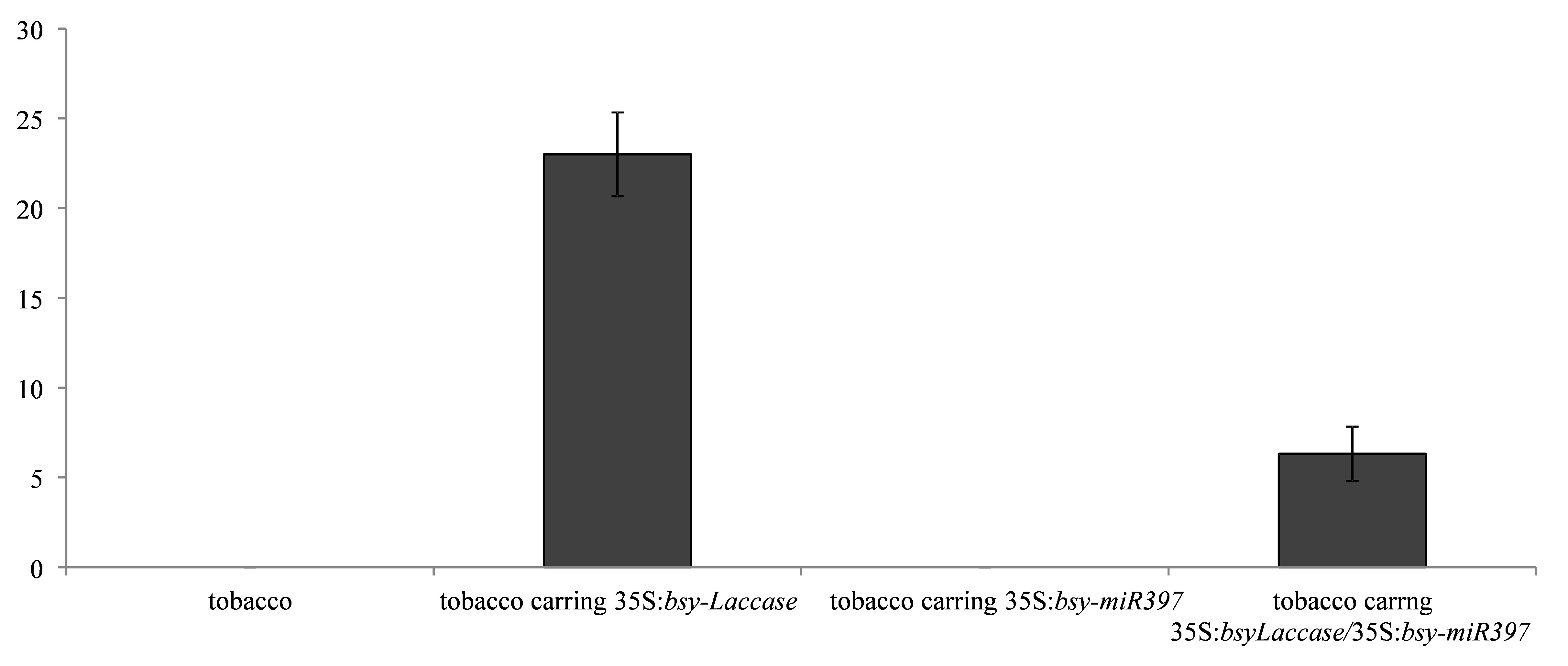


**Figure S1.**

**Supporting** **Tables**

**Table S1.** *Bsy-mi397* primers used for qRT-PCR analysis

| miRNA | RT primer (5′–3′) | Forward primer (5′–3′) |
| --- | --- | --- |
| *bsy-miR397* | GTCGTATCCAGTGCAGGGTCCGAGGTATTCGCACTGGATACGACTTCATC | GCTGGATTGAGTGCAGCGTT |

**Table S2.** Primers for *bsy-Laccase* genes used in qRT-PCR analysis

| Gene | Direction | Sequence (5′–3′) | Amplicon size (bp) |
| --- | --- | --- | --- |
| *bsy-Laccase* | Forward | TCGTTGAAATTTCCGAGACC | 243 |
|  | Reverse | CAAAGGCGTCTACGAGGAAG |  |

**Table S3.** Primers for *bsy-Laccase* genes used in amplifying overall length by reverse transcription PCR

| Gene | Direction | Sequence (5′–3′) | Amplicon size (bp) |
| --- | --- | --- | --- |
| *bsy-Laccase* | Forward | CGGAATTCATGCACCTACAGGCACG | 1,740 |
|  | Reverse-stop | CGGGATCCCTAACACTTTGGCATGATGCTAGG |  |

**Table S4.** Primers used in amplifying amiR-*bsy-miR397*

| Primers | Sequence (5′–3′) |
| --- | --- |
| bsy-397I | gaATTGAGTGCAGCGTTGATGAAtctctcttttgtattcc |
| bsy-397 II | gaTTCATCAACGCTGCACTCAATtcaaagagaatcaatga |
| bsy-397 III | gaTTAATCAACGCTGGACTCAATtcacaggtcgtgatatg |
| bsy-397 IV | gaATTGAGTCCAGCGTTGATTAAtctacatatatattcct |
| A | CTGCAAGGCGATTAAGTTGGGTAAC |
| B | GCGGATAACAATTTCACACAGGAAACAG |

**Table S5.** Primers for amplification of rice TFs and oxidase/peroxidase genes used in qRT-PCR analysis

| Rice Gene | Direction | Sequence (5′–3′) | Amplicon size (bp) |
| --- | --- | --- | --- |
| *Os-L-ascorbate oxidase* | Forward | GGCGAGACCTTCACCTACAG | 179 |
|  | Reverse | CACCAGTCCATGAGGAGGAC |  |
| *Os-Ubiquinol oxidase 1* | Forward | TGCTCAGCTCTGCAGTTGTT | 170 |
|  | Reverse | TTCCATGCACACATGAATCC |  |
| *Os-Peroxidase 1* | Forward | ACTCCTTCACCAATCGCATC | 197 |
|  | Reverse | GTTTGTCAGCCCGACGTAGT |  |
| *Os-Peroxidase 66* | Forward | CGTCACGACTGAGGAGTTCA | 161 |
|  | Reverse | GATCCTCAGCTGGACTTTGC |  |
| *Os-ARF5* | Forward | AAGCAACACCATTGCTACCC | 198 |
|  | Reverse | TTGAACAAGAGGGCATTTCC |  |
| *Os-ARF8* | Forward | GTCTTCAACTTCGGCGACTC | 185 |
|  | Reverse | GACTGCAGGTAAGGGCTCAG |  |
| *Os-MYB2* | Forward | TCATGAATCACATCGCCAAG | 172 |
|  | Reverse | TGCAGCTGAATGATGAGGTC |  |
| *Os-MYB39* | Forward | AGAAGGAGGGGCTCAAGAAG | 224 |
|  | Reverse | GTGGAGCTGGATGATGGTCT |  |
